# Epidemiology and transmission dynamics of COVID-19 in two Indian states

**DOI:** 10.1126/science.abd7672

**Published:** 2020-09-30

**Authors:** Ramanan Laxminarayan, Brian Wahl, Shankar Reddy Dudala, K. Gopal, Chandra Mohan B, S. Neelima, K. S. Jawahar Reddy, J. Radhakrishnan, Joseph A. Lewnard

**Affiliations:** 1 Center for Disease Dynamics, Economics and Policy, New Delhi, India.; 2 Princeton Environmental Institute, Princeton University, Princeton, NJ, USA.; 3 Department of International Health, Johns Hopkins Bloomberg School of Public Health, Baltimore, MD, USA.; 4 International Vaccine Access Center, Johns Hopkins Bloomberg School of Public Health, Baltimore, MD, USA.; 5 Department of Community Medicine, Government Medical College, Kadapa, Andhra Pradesh, India.; 6 Animal Husbandry, Dairying and Fisheries Department, Government of Tamil Nadu, Chennai, Tamil Nadu, India.; 7 Backward Classes, Most Backward Classes, and Minorities Welfare Department, Government of Tamil Nadu, Chennai, Tamil Nadu, India.; 8 Department of Community Medicine, Guntur Medical College, Guntur, Andhra Pradesh, India.; 9 Department of Health, Family Welfare, and Medical Education, Government of Andhra Pradesh, Amaravati, Andhra Pradesh, India.; 10 Health and Family Welfare Department, Government of Tamil Nadu, Chennai, Tamil Nadu, India.; 11 Division of Epidemiology, School of Public Health, University of California, Berkeley, CA, USA.; 12 Center for Computational Biology, College of Engineering, University of California, Berkeley, CA, USA.

## Abstract

Although most cases of coronavirus disease 2019 (COVID-19) have occurred in low-resource countries, little is known about the epidemiology of the disease in such contexts. Data from the Indian states of Tamil Nadu and Andhra Pradesh provide a detailed view into severe acute respiratory syndrome coronavirus 2 (SARS-CoV-2) transmission pathways and mortality in a high-incidence setting. Reported cases and deaths have been concentrated in younger cohorts than would be expected from observations in higher-income countries, even after accounting for demographic differences across settings. Among 575,071 individuals exposed to 84,965 confirmed cases, infection probabilities ranged from 4.7 to 10.7% for low-risk and high-risk contact types, respectively. Same-age contacts were associated with the greatest infection risk. Case fatality ratios spanned 0.05% at ages of 5 to 17 years to 16.6% at ages of 85 years or more. Primary data from low-resource countries are urgently needed to guide control measures.

## Epidemiology in southern India

By August 2020, India had reported several million cases of severe acute respiratory syndrome coronavirus 2 (SARS-CoV-2), with cases tending to show a younger age distribution than has been reported in higher-income countries. Laxminarayan *et al.* analyzed data from the Indian states of Tamil Nadu and Andhra Pradesh, which have developed rigorous contact tracing and testing systems (see the Perspective by John and Kang). Superspreading predominated, with 5% of infected individuals accounting for 80% of cases. Enhanced transmission risk was apparent among children and young adults, who accounted for one-third of cases. Deaths were concentrated in 50- to 64-year-olds. Incidence did not change in older age groups, possibly because of effective stay-at-home orders and social welfare programs or socioeconomic status. As in other settings, however, mortality rates were associated with older age, comorbidities, and being male.

*Science*, this issue p. 691; see also p. 663

## Teaser

The epidemiology of SARS-CoV-2 in southern India shows marked differences from that observed in higher-income countries.

Severe acute respiratory syndrome coronavirus 2 (SARS-CoV-2), the virus that causes coronavirus disease 2019 (COVID-19), has spread rapidly around the world since emerging in Wuhan, China, in late 2019 (*1*). Our current understanding of COVID-19 comes largely from disease surveillance and epidemiologic studies undertaken during the early phases of the pandemic in China (*1*–*3*) and in the high-income countries of Europe (*4*, *5*) and North America (*6*–*8*). However, most confirmed cases of COVID-19 have now occurred in low- and middle-income countries (LMICs), where a substantial proportion of individuals may be at increased risk of severe outcomes and face barriers to accessing quality health services (*9*–*11*). Although multiple modeling studies have sought to assess how COVID-19 might affect individuals and communities in such settings (*12*–*14*), almost no primary studies of the transmission dynamics and clinical outcomes of COVID-19 in LMICs are available to validate these models and inform intervention strategies (*15*).

More than 1.3 billion people are at risk of SARS-CoV-2 infection in India, where concerns over COVID-19 have prompted large-scale containment strategies at the national, state, and local levels (*16*). The country’s first known COVID-19 case, documented on 30 January 2020, was an Indian national evacuated from China (*17*). Andhra Pradesh and Tamil Nadu are two states in the south of India whose 127.8 million residents collectively account for about 10% of the country’s total population. Although they are not the wealthiest states in India, Andhra Pradesh and Tamil Nadu are among the states with the largest health care workforces and public health expenditures per capita, and are known for their effective primary health care delivery models (*18*–*20*). Both states initiated rigorous disease surveillance and contact tracing early in response to the pandemic. Procedures include syndromic surveillance and SARS-CoV-2 testing for all individuals seeking care for severe acute respiratory illness or influenza-like illness at health care facilities; delineation of 5-km “containment zones” surrounding cases for daily house-to-house surveillance to identify individuals with symptoms; and daily follow-up of all contacts of laboratory-confirmed or suspected COVID-19 cases, with the aim of testing these individuals 5 to14 days after their contact with a primary case, irrespective of symptoms, to identify onward transmission (*21*, *22*). We analyzed comprehensive surveillance and contact-tracing data from these programs in an effort to understand transmission dynamics and clinical outcomes of COVID-19 in South India, and to provide insights into control of SARS-CoV-2 in similar LMIC settings.

## Expansion of SARS-CoV-2

In India, surveillance of COVID-19 was initiated with airport screening for severe acute respiratory infection, especially for travelers from China. Tamil Nadu further instituted thermal and clinical screening at land borders with other states on 4 March 2020. Nationwide, testing was initially prioritized for symptomatic individuals with history of travel or contact with a confirmed COVID-19 case within the previous 14 days, and was expanded to include all symptomatic individuals and asymptomatic contacts of confirmed cases in states between 20 and 28 March 2020. We detail the timeline of changes in surveillance practices at federal and state levels in the supplementary materials.

**Fig. 1:**
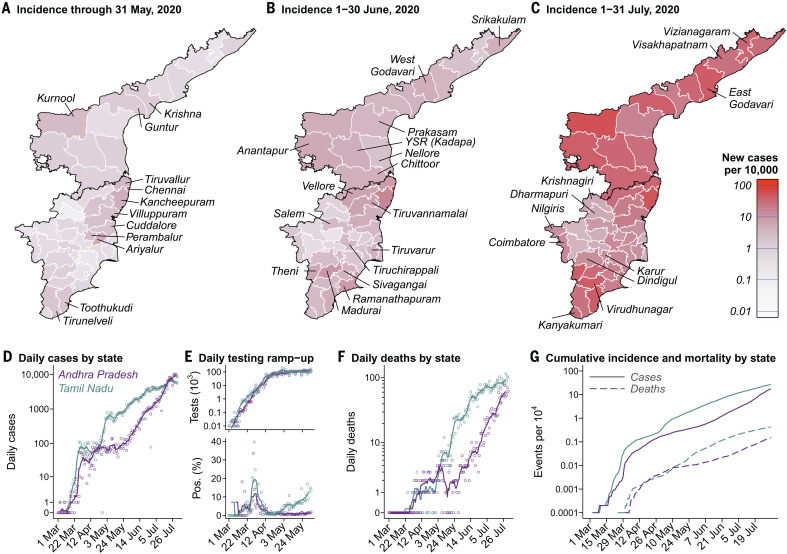
Incidence over time and across districts in Tamil Nadu and Andhra Pradesh. (**A** to **C**) Red shading of regions on the choropleth map indicates higher incidence over each period: 1 March to 31 May 2020 (A), 1 to 30 June 2020 (B), and 1 to 31 July 2020 (C). Districts are plotted according to 2019 administrative boundaries and do not reflect the recent bifurcation of Tirunelveli, Villuppuram, Vellore, and Chengalpattu districts. (**D**) Cases detected each day in each state (points) and 7-day moving averages (lines). Cases are aggregated by testing date; data are plotted in blue and lavender for Tamil Nadu and Andhra Pradesh, respectively, for all figure panels. (**E**) Diagnostic tests conducted each day (top) and the proportion of tests yielding positive results (bottom) for the period March through May 2020, when districts reported comprehensive testing information to the state governments. Points and lines indicate daily counts and 7-day moving averages, respectively. The high proportion of positive tests from late March to mid-April, while case number remained relatively stable, may indicate a period during which cases were undercounted because of limited testing capacity. (**F**) Daily deaths in the two states. Points and lines indicate daily counts and 7-day moving averages, respectively. (**G**) Cumulative incidence (solid lines) and mortality (dashed lines) per 10,000 population.

Tamil Nadu and Andhra Pradesh each recorded their first laboratory-confirmed COVID-19 cases on 5 March. Under-ascertainment of cases during March and early April was likely due to limited testing availability and testing algorithms. The proportion of tests yielding positive results peaked at 39.7% in Tamil Nadu and 33.5% in Andhra Pradesh on 30 and 31 March 2020, respectively, when the daily number of tests performed was low in the two states (range, 379 to 469 tests; Fig. 1). Throughout early April, increases in the number of tests performed daily coincided with a reduction in the proportion of tests yielding positive results. Our analyses include data collected through 1 August, at which time Tamil Nadu and Andhra Pradesh had identified 263,330 and 172,209 cases, respectively (table S1). (Because testing and contact tracing constitute routine public health activities, data collection was not governed by an institutional review board.)

The earliest clusters of locally acquired cases emerged in March in Chennai and surrounding coastal districts of eastern Tamil Nadu. Of all districts, Chennai ultimately experienced the highest cumulative incidence of COVID-19, totaling 102,199 cases (204.6 per 10,000 population) by 1 August 2020. An outbreak beginning on 28 April caused 1142 cases by 15 May in the adjoining districts of Ariyalur, Cuddalore, Perambalur, and Villuppuram in Tamil Nadu; thereafter, few cases were identified in these districts until early June (fig. S1). Although limited in March and April, incidence in southern districts of Tamil Nadu surrounding Madurai increased during June and reached rates commensurate with incidence in the northern districts of Chennai, Kancheepuram, and Tiruvallur by 1 August, with one to four new positive detections per 10,000 population daily. Similar increases in incidence occurred throughout all districts of Andhra Pradesh in June, where the numerical and geographic extent of cases remained limited during April and May despite similar levels of testing relative to Tamil Nadu.

Statewide estimates of the time-varying reproduction number *R*_t_, describing the number of secondary infections that each infected individual would be expected to generate (*23*), declined from a range of 1.7 to 3.0 in Tamil Nadu and 1.4 to 4.3 in Andhra Pradesh over the period 10 to 23 March to a range of 1.0 to 1.3 in both states by the third week of the initial countrywide lockdown (fig. S3). Expansions in testing over this same period, however, are likely to bias analyses of changes in *R*_t_ over time (*24*). Estimates of *R*_t_ held in the range 1.1 to 1.4 from 15 May onward within both states, although incidence trajectories differed over time by district (fig. S1), likely reflecting changes in both the uptake and enforcement of social distancing interventions as well as the effectiveness of contact-tracing efforts.

## Contact tracing

Contact-tracing efforts in the states reached 3,084,885 known exposed contacts of confirmed cases by 1 August 2020 (table S2); individual-level epidemiological data on cases and contacts, as well as laboratory test results, were available from 575,071 tested contacts of 84,965 confirmed cases. Traced contacts tended to be younger and were more often female than their linked index cases (table S3). Additionally, test-positive individuals identified through contact tracing were, on average, 1.3 years (bootstrap 95% confidence interval, 1.1 to 1.5 years) younger and 4.5% (3.7 to 5.4%) less likely to be male than the overall population of COVID-19 cases in the two states (table S4). Because studies in other settings have shown the risk of symptomatic disease to be higher among older age groups and among males (*25*), these findings may indicate the identification of less-severe infections through active case finding.

**Fig. 2:**
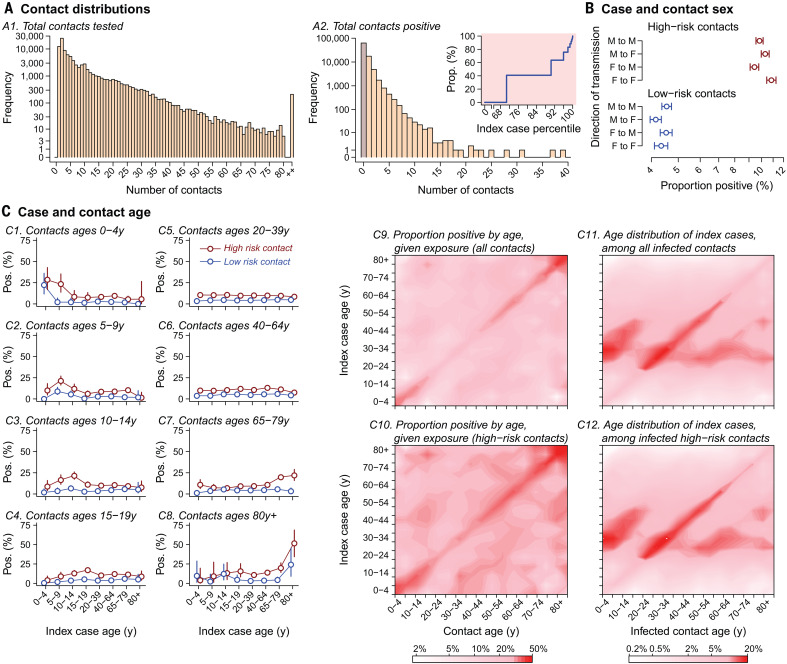
Analyses of contact-tracing data for 575,071 tested contacts of 84,965 infected individuals from whom test results were available, together with individual-level detailed epidemiological data on exposed contacts and index cases. (**A**) Left: Distribution of the number of contacts traced for each index case in Tamil Nadu and Andhra Pradesh, binning values ≥80 (0.2%). Right: Number of positive contacts traced from each index case. The inset shows the cumulative attributable proportion of secondary infections (*y* axis) associated with quantiles (*x* axis) of the distribution of the number of positive contacts traced per index case; percentiles 0 and 100 indicate index cases with the fewest and the most positive contacts identified, respectively. (**B**) Adjusted estimates from Poisson regression models addressing the proportion of female and male contacts with a positive result among those who were known to be exposed to female and male index cases; models further control for case and contact age groups (interacted) and for state. We stratify for high-risk and low-risk contacts, as defined in table S6. Points and lines indicate mean estimates and 95% confidence intervals. (**C**) Proportion of contacts with a positive test result stratified by case and contact age, for high-risk and low-risk contacts. At right, contour plots indicate the proportion of exposed contacts with a positive test result by case and contact age for all contacts and high-risk contacts on a choropleth scale; see table S8 for raw counts. Positive test results among tested, exposed contacts are interpreted as evidence of probable transmission from the index case. Also plotted are the age distributions of index cases for all infected contacts and for infected high-risk contacts.

The mean number of contacts tested per index case was 7.3 (interquartile range, 2 to 9), and 0.2% of index cases were linked to >80 tested contacts (range, 1 to 857; Fig. 2A). Numbers of contacts tested varied by district, and the geographic distribution of index cases included in our analyses did not necessarily reflect the geographic distribution of all reported cases (table S5). No positive contacts were identified for 70.7% of index cases for whom reliable contact-tracing data, including test results, were available (Fig. 2A). The distribution of the number of positive contacts linked to each index case was heavily right-skewed, and we estimated a negative binomial dispersion parameter for the distribution of the number of infected contacts traced to each index case of 0.51 (95% confidence interval, 0.49 to 0.52). On average, 9.2 contacts were tested for each index case with ≥1 contact identified, as compared to 5.7 contacts tested for each index case without positive contacts identified (two-sided bootstrap *P* < 0.001; fig. S4). Although our analysis is limited in that it does not necessarily capture all secondary infections (e.g., among contacts who were not reported), these observations are consistent with the presence of superspreading related to differences in individual contact patterns (*26*).

Assuming that test-positive contacts were infected by the index case to whom they were traced, we estimated that the overall secondary attack rate (or risk of transmission from an index case to an exposed contact) was 10.7% (10.5 to 10.9%) for high-risk contacts, who had close social contact or direct physical contact with index cases without protective measures, and 4.7% (4.6 to 4.8%) for low-risk contacts, who were in the proximity of index cases but did not meet these criteria for high-risk exposure (tables S6 and S7). Data on exposure settings, available for 18,485 contacts of 1343 index cases, revealed considerable differences in transmission risk associated with differing types of interaction. Secondary attack rate estimates ranged from 1.2% (0.0 to 5.1%) in health care settings to 2.6% (1.6 to 3.9%) in the community and 9.0% (7.5 to 10.5%) in the household. Among 78 individuals with high-risk travel exposures—defined as close proximity to an infected individual in a shared conveyance for ≥6 hours—we estimated a secondary attack rate of 79.3% (52.9 to 97.0%).

Whereas secondary attack rate estimates did not differ considerably with respect to the sex of cases and their contacts (Fig. 2B), analyses stratified by case and contact age identified the highest probability of transmission, given exposure, within case-contact pairs of similar age (Fig. 2C and table S8). These patterns of enhanced transmission risk in similar-age pairs were strongest among children aged 0 to 14 years and among adults aged ≥65 years, and may reflect differences in the nature of intragenerational and intergenerational social and physical interactions in India (*27*). Nonetheless, the greatest proportion of test-positive contacts within most age groups were exposed to index cases aged 20 to 44 years (Fig. 2C, fig. S5, and table S8). Serological surveys in other settings have demonstrated that case-based surveillance may lead to underestimation of SARS-CoV-2 infection prevalence among children (*28*, *29*); therefore, it remains crucial to establish whether the role of children in transmission is underestimated in studies such as ours using case-based surveillance to identify index infections.

## Mortality among COVID-19 cases

**Fig. 3:**
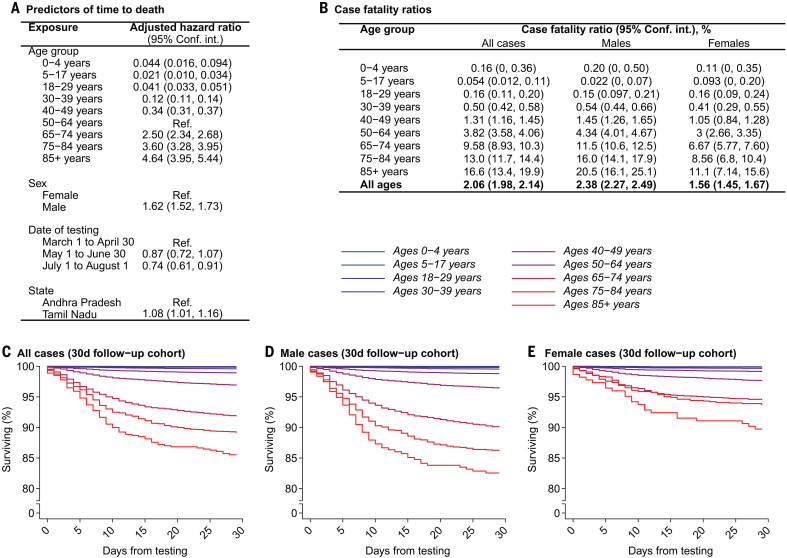
Mortality among confirmed COVID-19 cases. (**A**) Adjusted hazard ratios for mortality by 1 August 2020 estimated via Cox proportional hazards models including all confirmed cases. Exposures designated “Ref.” indicate the referent group for hazard ratio calculation. (**B**) Absolute case fatality risk estimates obtained via bootstrap resampling of individuals with confirmed infection by 1 July 2020. (**C** to **E**) Survival probabilities by age within this cohort over the 30-day period after testing, plotted for all cases (C), male cases (D), and female cases (E). Blue-to-red coloration aligns with younger-to-older age group, for strata as defined in the above tables. Age bins were selected on the basis of reporting of U.S. COVID-19 surveillance data (Fig. 4).

In a subcohort of 102,569 cases in Tamil Nadu and 22,315 cases in Andhra Pradesh who tested positive at least 30 days before the end of the study follow-up period, the overall case fatality ratio was 2.06% (1.98 to 2.14%; Fig. 3). Age-specific estimates ranged from 0.05% (0.012 to 0.11%) at ages 5 to 17 years to 16.6% (13.4 to 19.9%) at ages ≥85 years. Risk of death was higher among male cases than among female cases overall, and the magnitude of this difference widened in the oldest age groups. Higher mortality in older age groups and among males has similarly been observed in high-income settings (*1*–*7*, *30*–*32*).

Half of the cases ascertained before death in Tamil Nadu and Andhra Pradesh succumbed within ≤6 days of testing (interquartile range, 3 to 12 days), and 1042 fatal cases (18.2% of 5733 observed) were identified either ≤24 hours before death or posthumously. Our estimates of time to death in Tamil Nadu and Andhra Pradesh are below what has been observed internationally: In the United States, median time to death from the date of hospital admission was 13 days (*8*), and the World Health Organization estimated that time to death after onset of symptoms could range from 2 to 8 weeks on the basis of data from China (*33*). Our observations likely indicate that a substantial proportion of patients in Tamil Nadu and Andhra Pradesh are diagnosed late in their disease course, although differences in patients’ health status, health care systems capacity, and approaches to end-of-life care may also contribute to variation in time to death.

In a survival analysis of the full cohort, mortality by 1 August 2020 was independently associated with older age, with stepwise increases in the adjusted hazard ratio of time to death for each successive age group besides children aged 0 to 4 years, consistent with our estimates of the case fatality ratio (Fig. 3). Additional predictors of mortality included being male [adjusted hazard ratio, 1.62 (1.52 to 1.73) compared with being female], receipt of a test early in the epidemic [0.87 (0.72 to 1.07) for being tested between 1 May and 30 June, 0.74 (0.61 to 0.91) for being tested between 1 July and 1 August, both relative to testing between 1 March and 30 April], and state of residence [1.08 (1.01 to 1.16) for residents of Tamil Nadu compared with those in Andhra Pradesh].

Among decedents in the two Indian states, the most prevalent comorbid conditions were diabetes (45.0%), sustained hypertension (36.2%), coronary artery disease (12.3%), and renal disease (8.2%; table S9). Although prevalence of any comorbidity was highest among decedents at older ages, this pattern differed across conditions; diabetes was most prevalent among decedents aged 50 to 64 years, and liver disease and renal disease were most prevalent in fatal cases at ages 0 to 17 years and 18 to 29 years, respectively. At least one comorbid condition was noted among 62.5% of fatalities, in comparison to 22% of fatalities in the United States as of 30 May 2020 (*34*).

## Epidemiological comparison to high-income settings

**Fig. 4:**
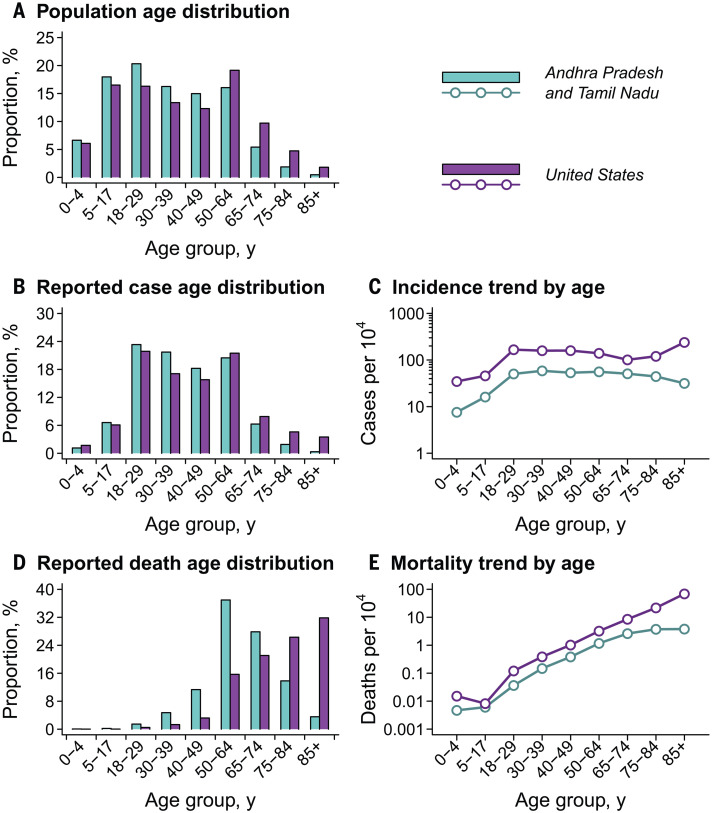
Demographic comparison of populations, cases, and deaths for Tamil Nadu and Andhra Pradesh versus the United States. (**A**) Age distribution of the population of Tamil Nadu and Andhra Pradesh (blue) against that of the U.S. population (purple) for comparison; underlying data are shown in table S10. Estimates are census extrapolations for the year 2020 in both settings. (**B**) Age distribution of cases. (**C**) Cumulative incidence of COVID-19 by age. (**D**) Age distribution of deaths. (**E**) Cumulative COVID-19 mortality by age. Data for the United States include all cases and deaths reported by 21 August 2020 (*35*).

Cases in Tamil Nadu and Andhra Pradesh showed a younger age distribution than cases reported in the United States as of 21 August 2020 (Fig. 4) (*35*). Comparison of cumulative COVID-19 incidence across ages showed that the observed differences surpassed expectations based on population age distributions alone, as signaled by the absence of parallel trends in age-specific incidence (table S10). Although lower across all age groups in Tamil Nadu and Andhra Pradesh in comparison to the United States, age-specific COVID-19 incidence increased sharply in both settings between the 5- to 17-year and the 18- to 29-year age groups. Whereas incidence declined steadily at ages older than 30 to 39 years in the two Indian states, incidence increased at ages of ≥65 years in the United States.

In the two Indian states, only 17.9% of COVID-19 deaths occurring on or before 1 August 2020 were among individuals aged ≥75 years, compared with 58.1% of COVID-19 deaths in the United States (Fig. 4 and table S10). Age-specific COVID-19 mortality was lower in Tamil Nadu and Andhra Pradesh than in the United States, consistent with the lower reported incidence of disease. Although COVID-19 mortality trended upward across ages in the two Indian states, mortality plateaued at ages of ≥65 years, in contrast to observations in the United States where COVID-19 mortality reached 69.6 deaths per 10,000 individuals aged ≥85 years; this observation was consistent with the relatively lower incidence of disease at the oldest ages within the two Indian states.

## Discussion

Our findings, based on comprehensive surveillance and contact-tracing data from the Indian states of Tamil Nadu and Andhra Pradesh, provide insight into the epidemiology of COVID-19 in resource-limited populations. Our analysis suggests substantial variation in individuals’ likelihood of transmitting: No secondary infections were linked to 71% of cases whose contacts were traced and tested. Although the role of children in transmission has been debated (*36*, *37*), we identify high prevalence of infection among children who were contacts of cases around their own age; this finding of enhanced infection risk among individuals exposed to similar-age cases was also apparent among adults. School closures and other nonpharmaceutical interventions during the study period may have contributed to reductions in contact among children. Nonetheless, our analyses suggest that social interactions among children may be conducive to transmission in this setting. Last, our analyses of fatal outcomes reveal an overall case fatality ratio of 2.1%. Even though our estimates of age-specific case fatality ratios are similar to those in other settings, such comparisons are limited by uncertainty in the proportion of infections ascertained as cases (*30*, *38*). Lower relative incidence of COVID-19 among older adults in Tamil Nadu and Andhra Pradesh has contributed to stark differences in the overall case fatality ratio and age distribution of decedents relative to observations in the United States and other high-income countries (*32*).

Several factors may contribute to our observation of limited COVID-19 incidence and mortality among older adults in Tamil Nadu and Andhra Pradesh. Imperfect surveillance systems may have contributed to under-ascertainment of cases among older adults, although this circumstance is unexpected given strong public and clinical awareness of COVID-19 and the predisposition of older adults to severe disease. Case-based surveillance may likewise underestimate attack rates among younger adult age groups in high-income settings (*28*, *29*). It is plausible that stringent stay-at-home orders for older Indian adults, coupled with delivery of essentials through social welfare programs and regular community health worker interactions, contributed to lower exposure to infection within this age group in Tamil Nadu and Andhra Pradesh. Our finding may also reflect survivorship bias if older adults in India are at disproportionately low risk for SARS-CoV-2 infection relative to the general population—for instance, as a result of higher socioeconomic status (*39*). Life expectancy at birth is 69 years in India, versus 77 years in China, 79 years in the United States, and 83 years in Italy and South Korea (*40*); as such, socioeconomic factors distinguishing individuals who survive to old age from the general population are likely more pronounced in India than in higher-income settings with longer average life expectancies (*41*, *42*).

Prospective testing of a large sample of exposed individuals through integrated active surveillance and public health interventions in Tamil Nadu and Andhra Pradesh provided an opportunity to characterize secondary attack rates as a function of both case and contact age, identify risk factors for transmission, and account for deaths outside of health care settings—a limitation of mortality surveillance in other settings (*30*, *43*, *44*). However, several limitations should be considered. The contact-tracing data analyzed included only 20% of all reported cases as index cases and represented only 19% of all contacts traced; case-finding efforts further varied by district and over time within Tamil Nadu and Andhra Pradesh. Contacts who complete testing and supply personal information to tracing teams may not have been representative of the full population. Another limitation was the lack of data on timing of exposure and onset of symptoms in relation to testing dates; this necessitated assumptions about the identification of true index cases. More robust temporal data would reduce the dependence on such assumptions, provide greater insight into the directionality of transmission, and reduce risk for misclassification of infection status among contacts with positive or negative results at the time of testing (*45*, *46*). The lack of temporal data also prevented us from estimating several epidemiologic parameters of interest. Current estimates of both the incubation period (~4 to 6 days) and the serial interval (~3 to 5 days) come from China (*1*, *47*–*51*). Several factors can modify the incubation period of respiratory viral infections, including the route of acquisition, the infectious dose, and the period of exposure to infected cases (*52*). The serial interval between successive infections is expected to be lower in high-transmission settings. Data allowing estimation of these parameters for SARS-CoV-2 in LMICs are needed to inform quarantine policies and other epidemic response efforts. Some true positives might have been misclassified as a result of imperfect test sensitivity, particularly among contacts tested as few as 5 days after exposure to a confirmed case. Imperfect test sensitivity has been attributed to inadequate sample collection procedures and low viral load in the upper respiratory tract, particularly for presymptomatic or asymptomatic cases (*53*). This limitation could lead to an overall underestimate of transmission risk within case-contact pairs. Finally, although comorbidity data collected as part of COVID-19 mortality surveillance revealed clinical and epidemiological attributes of fatal cases, the fact that such data were not collected for all diagnosed cases prevented inference of the contribution of comorbidities to fatal outcomes.

Surveillance and contact tracing are critical components of an effective public health response to COVID-19 (*54*, *55*). In our study, data generated by these activities within two states of South India provided key insights into the local epidemiology and transmission dynamics of SARS-CoV-2 without competing with emergency response activities for limited resources—a high priority in many LMICs where health workers and diagnostic equipment are already in short supply (*15*). Similar studies are necessary to inform the successful adoption of epidemic control measures in low-resource settings globally.

## Supplementary Materials

science.sciencemag.org/content/370/6517/691/suppl/DC1 Materials and Methods Figs. S1 to S5 Tables S1 to S10 MDAR Reproducibility Checklist
